# Supplementary material for: Multi-Observer Study on the Assessment of Pediatric Gonadal Tumors Using Higher Harmonic Generation Microscopy as Compared to Conventional Histology
Source: Cancers (Basel). 2025 May 12;17(10):1636. doi: 10.3390/cancers17101636 (PMC12110227; doi:10.3390/cancers17101636)
Supplement: Supplementary file 1 [file cancers-17-01636-s001.zip › cancers-3575542-supplementary.pdf]

Supplementary Data

S1: Overview of the question sheet

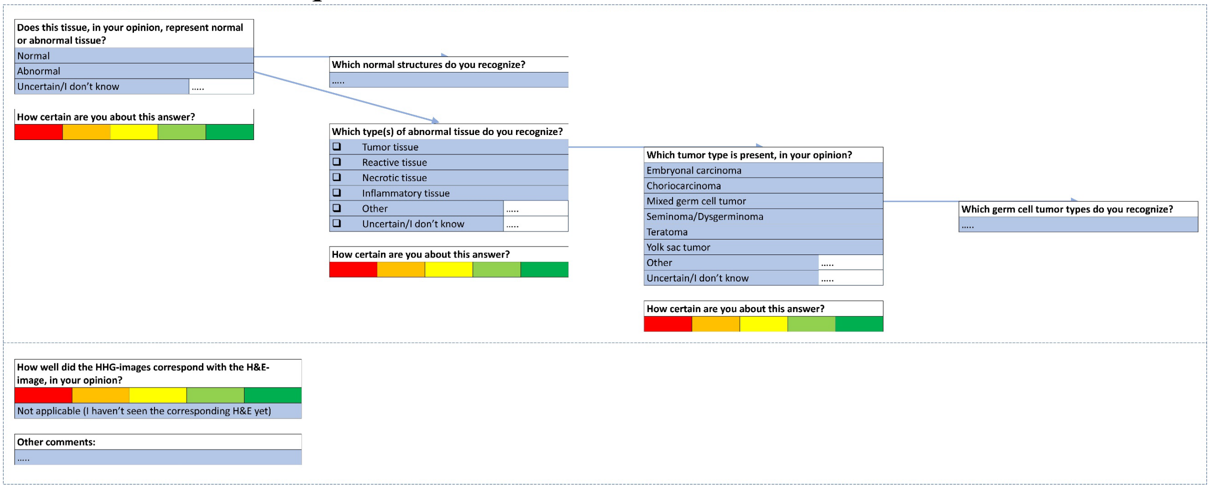

Supplementary Data 1 Overview of the questions that were asked in the Slide Score study. Most questions were dependent on the previous answer, which is visualized by arrows. These questions only appeared if a specific answer was given, to make sure that only the relevant questions were asked. Text fields represent questions where explanations could be given. Checkboxes represent questions where multiple answers could be selected. Most questions were obligatory, only the checkbox options and 'Other comments' were not obligatory. After each multiple-choice question, the uncertainty was given by very uncertain (red), uncertain (orange), neutral (yellow), certain (yellow green), very certain (green). Following the HHGM image, the pathologists received the corresponding H&E section with the same question sheet and could not see nor change their answers of the previous. In addition, pathologists assessed the correspondence between the HHGM images and the H&E image, using the following categories: very bad (red), bad (orange), neutral (yellow), good (yellow green) and very good (green). Since it was not possible to make separate question sheets for HHGM and H&E cases, the option `Not applicable, I haven't seen the corresponding histology yet' was added to these options.

S2: Overview of the cases

Supplementary Data 2 Overview of the cases that were included in this study. To include normal testis tissue in this study as well, four samples were taken from normal testis tissue sufficiently far away from the tumor. When two samples from the same patient were present, one of the samples was moved to the end of the Slide Score study.

Case	Location	Patient	Diagnosis
01	Lymph node	Patient 1	Teratoma
02	Testis	Patient 2	Normal reference
03	Testis	Patient 3	Normal reference
04	Testis	Patient 4	Mixed germ cell tumor
05	Ovary	Patient 5	Teratoma
06	Lymph node	Patient 6	Dysgerminoma metastasis
07	Testis	Patient 7	Embryonal carcinoma
08	Ovary	Patient 8	Follicle cyst
09	Ovary	Patient 9	Healthy
10	Testis	Patient 10	Teratoma
11	Testis	Patient 11	Leydig cell tumor
12	Ovary	Patient 12	Necrosis
13	Testis	Patient 13	Teratoma
14	Lymph node	Patient 14	Teratoma
15	Testis	Patient 15	Yolk sac tumor
16	Testis	Patient 16	Seminoma
17	Ovary	Patient 17	Teratoma
18	Testis	Patient 18	Normal reference
19	Ovary	Patient 19	Mucinous cystadenoma
20	Abdomen	Patient 20	Mixed germ cell tumor
21	Testis	Patient 21	Sertoli cell tumor
22	Neck/throat	Patient 22	Teratoma
23	Lymph node	Patient 1	Teratoma
24	Testis	Patient 2	Rhabdomyosarcoma
25	Testis	Patient 3	Mixed germ cell tumor
26	Ovary	Patient 5	Teratoma
27	Testis	Patient 18	Rhabdomyosarcoma
28	Testis	Patient 21	Normal reference

S3: Pathologists’ assessment for normal testis

Supplementary Data 3 Pathologists’ assessment for the normal testis cases. Each case consists of an HHGM part (upper row) and an H&E part (lower row). The columns P1-P5 represent the five pathologists in random order. Assessment of normal (N), tumor (T), reactive (R), inflammatory (I), necrotic (NC), other abnormal tissue (O), abnormal but uncertain which type (A?), or uncertain (?). ST = seminiferous tubules. Colors are used for a better visualization of non-tumor (green), tumor (red), GCNIS (orange) and uncertain (white), and are also based on the additional comments. For the purpose of this study, GCNIS is considered as non-tumor. The consensus was determined as the majority opinion (≥ 3 pathologists in agreement regarding tumor or non-tumor). The consensus was used to calculate the diagnostic characteristics.

Case	P1		P2		P3		P4		P5		Consensus	Tumor Diagnosis
Case 02 Testis Normal	N	Albuginea and the testicular parenchyma with ST	N	ST, interstitium (Leydig cells)	N	Testicular tubules, leydig cells	N	Prepubertal testicular parenchyma	O	Seminiferous cords appear irregularly shaped, raising the question of DSD. Perhaps Leydig cell hyperplasia as well.	HHG	
	N	Albuginea and prepubertal testis (ST)	N	ST, interstitium with Leydig cells	N	Prepubertal testicular tubules	T	GCNIS. I suspected there may be GCNIS in the HHG, but was hesitant... The H&E reveals is clearly.	T	GCNIS	H&E	
Case 03 Testis Normal	T	Sem/Dys	T	Sem/Dys	N	Testicular tubules, leydig cells, fibrosis, vessels	O	Dysgenetic pubertal testicular parenchyma. The testicular cords/tubules are dysmorphic and the interstitial space is expanded.	T	Sem/Dys	HHG	Sem/Dys
	N	Normal testicular parenchyma. Leydig cells and ST	N	ST (spermatogenesis), interstitium with Leydig cells	N	Testicular tubules with spermatogenesis	O	Testicular parenchyma with partial spermatogenesis and areas of GCNIS. The HHG does not show clearly the spermatogenesis.	N	ST with spermatogenesis, interstitial Leydig cells	H&E	
Case 18 Testis Normal	N	ST, Leydig cells in the interstitium	N	ST, interstitium	N	Normal postpubertal testis with spermatogenesis	N	Testicular parenchyma	N	ST, interstitium	HHG	
	?	Normal testicular background with reactive changes vs suspicious for GCNIS	N	Testicular parenchyma, ST	T, O	GCNIS	T, O	GCNIS	T	GCNIS	H&E	
Case 28 Testis Normal	N	ST	N	ST, interstitium	N	ST	T	GCNIS?	T	Sem/Dys	HHG	
	N	ST	N	ST, interstitium	N	ST	T	GCNIS	N	ST with focal spermatogenesis, interstitium with Leydig cells. I have a hard time recognizing normal seminiferous tubules in the HHG images. They appear packed to me, raising suspicious for intratubular seminoma. Hopefully with additional training I would be able to recognize them for what they are.	H&E	

S4: Pathologists’ assessment for nontumoral ovary

Supplementary Data 4 Pathologists' assessment for the nontumoral ovary cases.

Case	P1		P2		P3		P4		P5		Consensus	Tumor Diagnosis
Case 08 Ovary Follicle Cyst	?	I am not sure about the green part in the center. Could there be a cystic lesion here? The orange part could be just normal ovarian stroma.	R		?	Difficult to distinguish between normal and abnormal	R, A?	There appears to be extensive fibrosis	R, I		HHG	
	?	Normal ovarian parenchyma. Tubules in the center - disorder of sex development, ovotestis?	O	Streak gonad	T	may be sertoli cells/tumour	N	Ovarian parenchyma	R		H&E	
Case 09 Ovary Healthy	N	Ovarian parenchyma / stroma and vessel	N	Fibrous stroma, blood vessels	N	Normal ovarian tissue, may be fibroma	N	Ovarian parenchyma	N	Ovarian stroma	HHG	
	O	Fibrosis	A?	Fibrous tissue with reactive fibro/myofibroblasts; fibromatosis?	T	Fibroma	T	Ovarian fibroma/ sclerosis of the ovary	N	Ovarian stroma	H&E	
Case 12 Ovary Necrosis	R, I		N	Fibroconnective tissue, blood vessels	T	Ter	T	I cannot recognize a pattern...	R		HHG	
	R, I, O	Fibrosis, calcification, oedema. Post-chemotherapy effects?	R		R		T	Low cellularity tumor, likely benign. Possible myxoma.	R		H&E	

S5: Pathologists’ assessment for teratomas

Supplementary Data 5 Pathologists’ assessment for the teratoma cases

Case	P1		P2		P3		P4		P5		Consensus	Tumor Consensus
Case 01 Lymph Node Ter	O	Muscular tissue (striation?), fibrosis	R		N	Muscle connective tissue, vessels	N	Skeletal (striated) muscle, collagen	N	Skeletal muscle, aponeurosis?	HHG	
	O	Skeletal muscle and glia (?) could be a somatic type malignancy arising in a germ cell tumor?	T	Likely teratoma but only see in this section tissue from one embryonic layer	N	Muscle, fibrosis, connective tissue, vessels	T, R	Ter: The H&E reveals brain tissue, surrounded by skeletal muscle. The HHG image does not allow to recognize brain tissue.	T, O	Ter: Brain /glial tissue	H&E	Teratoma
Case 05 Ovary Ter	T	Ter: Cartilage and squamous epithelium?	T	Ter	T	Ter	T	Ter	T	Ter	HHG	Teratoma
	T	Ter	T	Ter	T	Ter	T	Ter	T	Ter	H&E	Teratoma
Case 10 Testis Ter	A?	Tissue near the spermatic cord? Rete testis?	N	Skeletal muscle, fibroconnective tissue with blood vessels, epididymis	N	Prepubertal testicular tubules	R		T	Ter	HHG	
	T, NC, I	Intestinal epithelium, mature. Could be part of a teratoma.	N	Colonic mucosa with submucosa and muscularis propria	T	Ter	T	Ter	R		H&E	Teratoma
Case 13 Testis Ter	T, R, O	Dense fibrosis. Calcification? Densely cellular process. Nucleoli. Tumor?	T	Sem/Dys	T	Ter	T	MGCT: Ter, possible EC	T	There are sheets of seemingly large, atypical cells, which I am not able to further characterize.	HHG	NO CONSENSUS
	T, O	Fibrosis. Spindle cell lesion / soft tissues - neural tumor? muscular tumor? Most of the H&E fragment is gone (torn apart)	A?	Very limited tissue, fibroconnective tissue	?	HHG images seem not to be the same tissues as in HE	R	Very different appearance between the HHG and H&E	R		H&E	
Case 14 Lymph Node Ter	T, O	Adipose tissue. Fibrosis. Bone formation? Teratoma in lymph-node?	N	Muscle, adipose tissue, lymphoid/cellular areas	T	Ter	A?	Possible areas of fibrosis	NC		HHG	
	R, I, O	Fibrosis. Post-chemotherapy effects?	O	Dense hypocellular fibrous tissue; differential including fibroma.	R, O	Tissue after therapy	R		NC, I		H&E	
Case 17 Ovary Ter	T, R	Can't match the features with any specific tumor type	N	Fibroconnective tissue, ovarian stroma with primordial follicles	N	N, I don't know	?	There appears to be a portion of ovarian parenchyma overlying a central area of fibrous tissue	R, I		HHG	
	R, I		O	At the edge there is neuropil, Ter?	T	May fibroma	O	Sclerotic ovary	R, I		H&E	
Case 22 Neck/Throat Ter	T, R, I	Ter	T, R	Ter	T	May be teratoma, not sure	T	EC	O	There seems to be epithelial tissue, possible cartilage, fat, fibrous connective tissue. Branchial cleft remnant?	HHG	Teratoma
	T, R, I	Ter	T	MGCT: Ter (95%), possible foci of YST (~5%)	T	Ter	T	Ter	T	Ter	H&E	Teratoma
Case 23 Lymph Node Ter	R, I, O	Blood vessels with blood. Cautery artifact.	R, I		T	Ter	A?	I cannot recognized the pattern	R		HHG	
	R, O	Fibrosis, skeletal muscle with cautery artifact, adipose tissue. Cellular proliferation (glial tissue, part of teratoma?)	T, R	Ter	T	Ter	T	Fibrous and glial tissue?	T	Glial tissue, CNS. Not sure of the etiology. Teratoma? The cellular (glial, neural) elements are really hard to see. It looks mostly like fibrous tissue/collagen in my opinion, similar to case 1.	H&E	Teratoma
Case 26 Ovary Ter	T, R	Ter	T	Ter	T	Ter	T	Ter	T	Ter	HHG	Teratoma
	T, NC	Ter	T	Ter	T	Ter	T	Ter	T	Ter	H&E	Teratoma

S6: Pathologists’ assessment for non-teratoma GCTs

Supplementary Data 6 Pathologists’ assessment for the non-teratoma cases.

Case	P1		P2		P3		P4		P5		Consensus	
Case 07 Testis EC	T, I, O	Vessels. calcification? cautery artifact? Can't find any features that make me favor a specific tumor type.	T	Sem/Dys	N	Epididymis	T	I believe this is tumor, but cannot recognize a specific pattern to make a dx.	T	Sem/Dys	HHG	NO CONSENSUS
	T, I	Sem/Dys with crushing artifacts? EC? Other tumor?	T	EC	T	EC	T	EC	T	EC	H&E	EC
Case 06 Lymph Node Sem/Dys	T	Sem/Dys	N	Reactive lymph node, lymphocytes, connective tissue	T	No good idea what it could be	N	Lymphoid follicles	T	EC	HHG	NO CONSENSUS
	T	Sem/Dys. Could notice the difference between the tumor cells and the lymphocytes on the HHG image.	T	Sem/Dys	T	maybe Sem/Dys, would perform immunohistochemistry	T, O	Residual lymphoid parenchyma. EC, metastatic to lymph node	T	EC	H&E	Sem/Dys
Case 16 Testis Sem/Dys	T, R, O	Fibrosis, densely cellular, prominent nucleoli. Lymphoma? Sem/Dys?	T	Sem/Dys	T	Sem/Dys	T, R	EC	T	EC	HHG	Sem/Dys or EC
	T	Sem/Dys	T	Sem/Dys	T	Sem/Dys	T	Sem/Dys	T	Sem/Dys	H&E	Sem/Dys
Case 15 Testis YST	T	YST	T	YST	?	Cannot interpret the tissue	T	EC	T	YST	HHG	YST
	T	YST	T	YST	T	YST	T	YST	T	YST	H&E	YST
Case 04 Testis MGCT	T	Sem/Dys	T	MGCT: Sem/Dys, EC?	T, R	Large nuclei with pleomorphism	T, R, NC	EC	T	EC	HHG	NO CONSENSUS
	T	MGCT: Ter, YST, Syncytiothroblast cells	T	MGCT: YST, EC, CC?	T	MGCT: EC, YST, CC	T	MGCT: EC, YST, foci of CC	T	YST	H&E	MGCT
Case 20 Abdomen MGCT	T	Ter, Possibly immature teratoma with rosettes? very cellular.	T	Uncertain but some areas are reminiscent of a Wilms tumor.	T	I have no idea what it could be	N	Neonatal ovarian parenchyma	T, NC	It appears epithelial, with nests and some tubules/cysts and possible areas of necrosis. Favor malignant.	HHG	NO CONSENSUS
	T	Ter	T	Ter	T	Ter	T	Ter	T	Ter	H&E	Teratoma
Case 25 Testis MGCT	T, R, I	Some large cells made me think of trophoblast cells. Perhaps a mixed tumor?	T	MGCT: Sem/Dys, maybe foci of YST and/or EC	T	Ter	T	EC	T	MGCT: It looks quite pleomorphic, with many large cells with high N:C ratio and prominent nucleoli, EC? There also seem to be some larger cells forming syncytia, CC?	HHG	NO CONSENSUS
	T	MGCT: EC, also foci of YST? The tissue on H&E is fragmented and looks different from the HHG image	T	MGCT: EC and YST	T	MGCT: Ter, YST, maybe EC (need IHC)	T	EC	T	MGCT: There is a carcinoma component and a spindle cell component. I am unsure of the type but suspect somatic malignancy arising from Ter.	H&E	MGCT

S7: Pathologists’ assessment for non-GCTs

Supplementary Data 7 Pathologists’ assessment for the non-germ cell tumor cases.

Case	P1		P2		P3		P4		P5		Consensus	
Case 19 Ovary Mucinous Cystadenoma	T, R	EC	T	Sem/Dys	N	Not sure	A?	I don't see a clear tumor...	T	It appears to be epithelial in origin but can't tell cell type.	HHG	NO CONSENSUS
	T, R	Mucinous cystadenoma?	T	Favor serous cystadenoma	T	Looks like an serous cystadenoma	T	Cystadenoma of the ovary	T	Serous cystadenoma. The epithelial component appeared much more solid/sheet-like rather than cystic in the HHG-image	H&E	Cystadenoma
Case 11 Testis Leydig Cell Tumor	T	Looks like a very compact tumor. I can't appreciate the cells well.	T	Sem/Dys	?	Bad picture quality	T	EC	T	Hematolymphoid?	HHG	NO CONSENSUS
	T	Leydig cell tumor?	T	Favor Leydig cell tumor	T	Leydig cell tumor	T	Stromal cell tumor (reminiscent of adrenal cortex)	T	YST	H&E	Leydig Cell Tumor
Case 21 Testis Sertoli Cell Tumor	R, I, A?	Enlarged ST, intratubular seminoma?	R, I		N	Normal testis and epididymis	T	Possible Leydig cell hyperplasia...	T	Sem/Dys	HHG	
	T, R	Sertoli cell tumor? Other?	T	favor a Sertoli cell tumor	T	May be undifferentiated sex cord stromal tumour	T	Possible stromal tumor	T	Sex cord stromal tumor? Sertoli cell	H&E	Sertoli Cell Tumor
Case 24 Testis RMS	R, I		T	EC	T	Not sure, may be teratoma	T	Spindle cell tumor?	T	Sheets of spindle to plump cells. Possible sarcoma, RMS?	HHG	NO CONSENSUS
	T	Spindle cell neoplasm, myxoid background. RMS?	T	Favor embryonal RMS	T	Not sure may be undifferentiated tissue in a teratoma	O	PMMTI? BCOR rearranged?	T	Favor embryonal RMS	H&E	RMS
Case 27 Testis RMS	T	Very densely cellular. Can't favor a specific tumor type.	T	Sem/Dys	T	Sem/Dys	T	EC	T	The tumor is composed of elongated and epithelioid/round cells with some large atypical cells. Not sure of the diagnosis..	HHG	NO CONSENSUS
	T	Spindle cell neoplasm. Rhabdomyoblasts. RMS? MPNST Triton? YST?	T	Favor embryonal RMS	T	YST	T	Spindle/clear cell tumors?	T	Favor embryonal RMS	H&E	RMS
